# Activity Patterns of St. Louis Encephalitis and West Nile Viruses in Free Ranging Birds during a Human Encephalitis Outbreak in Argentina

**DOI:** 10.1371/journal.pone.0161871

**Published:** 2016-08-26

**Authors:** Luis Adrián Diaz, Agustín Ignacio Quaglia, Brenda Salomé Konigheim, Analia Silvana Boris, Juan Javier Aguilar, Nicholas Komar, Marta Silvia Contigiani

**Affiliations:** 1 Laboratorio de Arbovirus—Instituto de Virología “Dr. J. M. Vanella”–Facultad de Ciencias Médicas–Universidad Nacional de Córdoba, Córdoba, Argentina; 2 Instituto de Investigaciones Biológicas y Tecnológicas–CONICET–Universidad Nacional de Córdoba, Córdoba, Argentina; 3 Centers for Diseases Control and Prevention, Fort Collins, Colorado, United States of America; University of California Davis, UNITED STATES

## Abstract

St. Louis encephalitis virus (SLEV) (*Flavivirus*) is a reemerging arbovirus in the southern cone of South America. In 2005, an outbreak of SLEV in central Argentina resulted in 47 human cases with 9 deaths. In Argentina, the ecology of SLEV is poorly understood. Because certain birds are the primary amplifiers in North America, we hypothesized that birds amplify SLEV in Argentina as well. We compared avian SLEV seroprevalence in a variety of ecosystems in and around Córdoba city from 2004 (before the epidemic) and 2005 (during the epidemic). We also explored spatial patterns to better understand the local ecology of SLEV transmission. Because West Nile virus (WNV) was also detected in Argentina in 2005, all analyses were also conducted for WNV. A total of 980 birds were sampled for detection of SLEV and WNV neutralizing antibodies. SLEV seroprevalence in birds increased 11-fold from 2004 to 2005. Our study demonstrated that a high proportion (99.3%) of local birds were susceptible to SLEV infection immediately prior to the 2005 outbreak, indicating that the vertebrate host population was primed to amplify SLEV. SLEV was found distributed in a variety of environments throughout the city of Córdoba. However, the force of viral transmission varied among sites. Fine scale differences in populations of vectors and vertebrate hosts would explain this variation. In summary, we showed that in 2005, both SLEV and to a lesser extent WNV circulated in the avian population. Eared Dove, Picui Ground-Dove and Great Kiskadee are strong candidates to amplify SLEV because of their exposure to the pathogen at the population level, and their widespread abundance. For the same reasons, Rufous Hornero may be an important maintenance host for WNV in central Argentina. Competence studies and vector feeding studies are needed to confirm these relationships.

## Introduction

St. Louis encephalitis virus (SLEV) (*Flavivirus*, *Flaviviridae*) is a reemerging arbovirus in the southern cone of South America (Argentina and Brazil) [1,2]. In 2005, an outbreak of St. Louis encephalitis in central Argentina resulted in 47 human cases with 9 deaths [1]. SLEV has been considered a serious public health threat since 1933, when it was first discovered during a large human encephalitis outbreak that initiated in Missouri, USA [3].

In North America, SLEV is maintained through transmission between *Culex* mosquito vectors and certain passeriform and columbiform birds. In North America, House Sparrow (Passeridae; *Passer domesticus*) and House Finch (Fringillidae; *Haemorhousmexicanus*) are thought to be principal amplifiers of SLEV in urban locations, with Mourning Dove (Columbidae; *Zenaidamacroura*) also contributing in rural regions [3].

In Argentina, the ecology of SLEV is poorly understood. The virus has been isolated from humans (Buenos Aires Province), *Culex* spp. mosquitoes (Córdoba and Santa Fe provinces) and wild rodents (Córdoba) [4,5]. SLEV has never been isolated from birds in Argentina. However, serological evidence indicates frequent infection of SLEV in Argentine birds [6]. In temperate and subtropical regions of Argentina, neutralizing antibodies were detected in a wide range of birds belonging to several families (Furnariidae, Columbidae, Tyrannidae, Fringillidae, Icteridae, Ardeidae and Cotingidae) [6].

In 2005, the first human encephalitis outbreak attributed to SLEV outside the USA took place in Córdoba Province in central Argentina [1], providing an opportunity to characterize the role of avian hosts in the ecology of SLEV in this locale. Because certain birds are the primary amplifiers in North America, we hypothesized that birds amplify SLEV in Argentina as well. Thus, we should detect a significant increase in viral activity in birds during an epidemic period compared to an enzootic period. To test this hypothesis, we compared avian SLEV seroprevalence in a variety of ecosystems in and around Córdoba city from 2004 (before the epidemic) and 2005 (during the epidemic). We also explored spatial patterns to better understand the local ecology of SLEV transmission. Because West Nile virus (Flaviviridae; WNV) was also detected in Argentina in 2005 [7, 8], and is closely related to SLEV, all analyses were also conducted for WNV.

## Material and Methods

### Study area and collection sites

**Fig 1:**
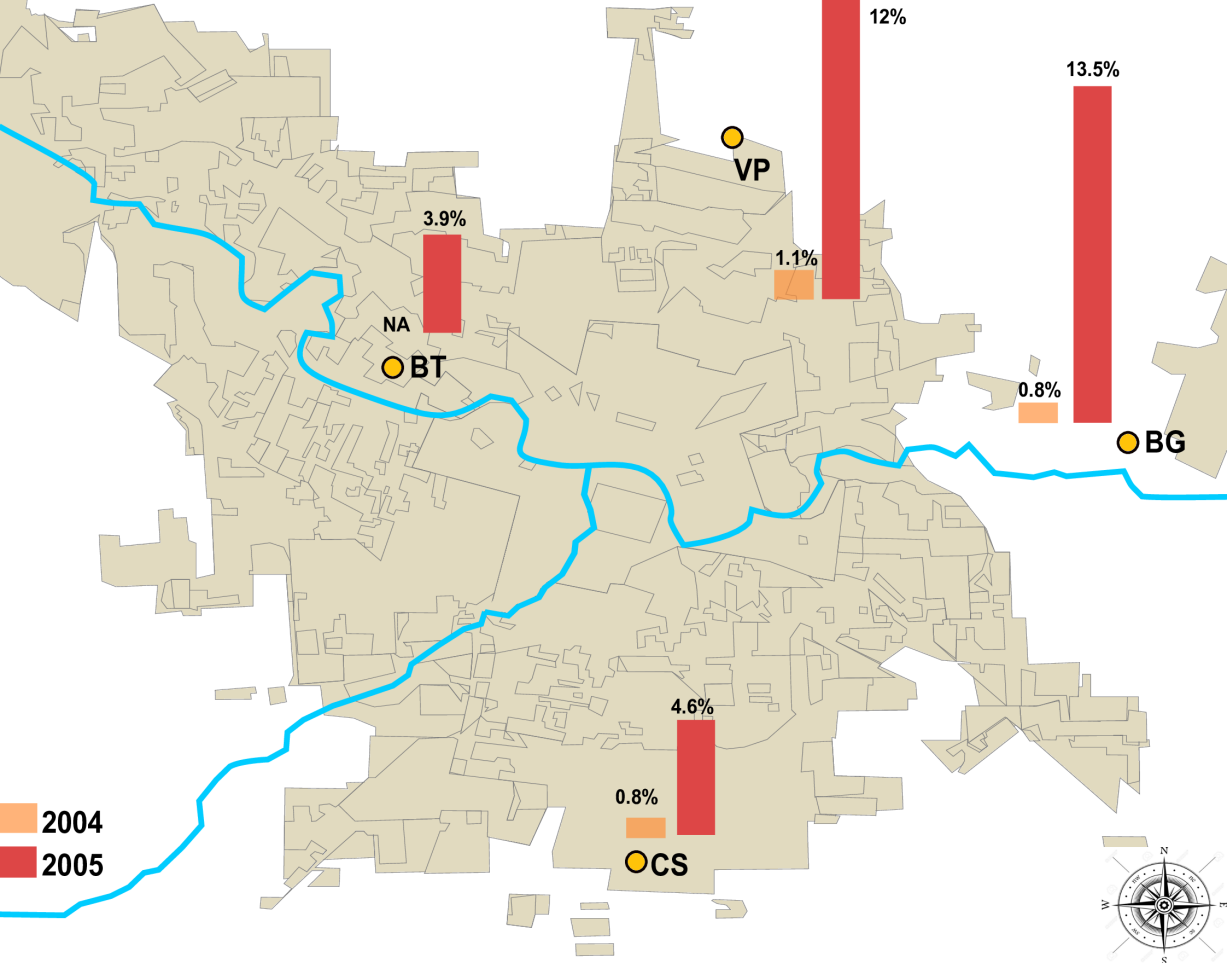
Geographic location of sampling sites in Córdoba city and neutralizing antibodies seroprevalence (%) in wild birds for St. Louis encephalitis virus and West Nile virus per site and year. BG: Bajo Grande, CS: Camino San Carlos, BT: Botanical Garden, VP: Villa Gran Parque. Bar graphs present seroprevalence values expressed as percentages (number of positive sera/number of analyzed sera).

Bird captures were carried out during summer and fall of 2004 and 2005 in 4 sites located in Córdoba city (31°24'30”S, 64°11'02” W) (Córdoba Province, Argentina) (Fig 1). This city of 1.3 million inhabitants is situated at 450 m above sea level, and encompasses an area of 576 km^2^ of which 37.2% is urbanized. The area belongs to the phytogeographic region called Espinal, Chaqueño Domain, or “Chaco Thorn-Forest” [9]. This region is characterized by semi-arid thorn scrub habitat but has been modified intensively by human activities (soy and fruit agriculture, cattle ranching, industrial activity). Isolated patches of natural habitat surrounding the city are comprised of shrub forest. The climate is temperate and semi-arid due to high evapotranspiration in spite of a relatively high precipitation level (750–800 mm) [10]. Four sampling sites were selected based upon accessibility, owners’ authorization and feasibility for mist net use; most of them are located in the periphery of the city of Córdoba (Fig 1):

1. Bajo Grande (BG) sewage treatment plant (31°23′38″ S; 64°04′36″ W). The site is surrounded by aquatic vegetation, reservoirs, low income human settlements and crop lands (vegetables and fruits). Vegetation is dominated by non-native chinaberry (*Melia azedarach*) and white mulberry (*Morus alba*) deciduous trees alternating with grasslands.
2. Botanical Garden (BT) (31°23′13″ S; 64°14′58″ W). The vegetation is characterized by patches of aquatic environments, grasslands, croplands and native thorn forest. It is surrounded by low and middle income human settlements.
3. Camino San Carlos (CS) farmland (31°28′46″ S; 64°09′26″ W). Two habitats present include native thorn forest dominated by *Acacia* sp., *Prosopis* sp., as well as cinacina (*Parkinsonia aculeata*), and grassland characterized by *Poa* sp., *Stipa* sp., Spanish needle (*Biden spilosa*) and introduced Chinese privet (*Ligustrum lucidum*) close to the family house.
4. Villa Gran Parque (VP) (31°19′55″ S; 64°10′28″ W). This vacant lot is surrounded by human settlements and croplands. The dominant habitat is grassland with Spanish needle, *Sorghum* sp., artichoke thistle (*Cynara cardunculus*) and alfalfa (*Medicago sativa*) with scattered white mulberry trees.

Permission for sampling work in Bajo Grande and Botanical Garden were obtained from the Municipality of Córdoba. Private landowner permission was obtained for study sites at Camino San Carlos and Villa Gran Parque.

### Bird captures and sample collection

Birds were captured with mist nets (AFO Mist Nets, Manomet, Inc., Manomet, MA, USA). Four mist nets per site were installed and operated at dawn and late afternoon. The bird capture was authorized by the Córdoba Province Environmental Agency. Captured birds were identified, weighed, sexed and aged when possible. Wild birds were marked with uniquely numbered aluminum leg bands. Before released, blood-sampled birds were rehydrated with sugar water. Whole blood was collected by jugular (most species) or brachial venipuncture (columbids). Birds that weighed less than 10 grams were not blood-sampled. Blood was placed into a centrifuge tube containing 0.45 mL or 0.9 mL (according to blood sample volume: 0.1 ml or 0.2 ml, respectively) of Minimum Essential Medium (MEM) for an approximate 1:10 serum dilution, held at ambient temperature for 20–30 min for coagulation and placed into coolers. At the laboratory, samples were centrifuged at 5,000g for 15 min for separation of serum. Sera were stored at -20°C. Prior to analysis, sera were heat-inactivated for 30 minutes at 56°C to inactivate non-specific inhibitors of virus neutralization.

### Ethics Statement

The bird capture was authorized by the Córdoba Province Environmental Agency (586869-053-107). Birds were handled following guidelines for the use of wild birds in research elaborated by the Ornithological Council (http://naturalhistory.si.edu/BIRDNET/documents/guidlines/Guidelines_August2010.pdf).

Field studies did not involve endangered or protected species. All blood sampling procedures were specifically approved as part of the obtaining field permit.

### Viral stocks preparation

Low-passage SLEV CbaAr-4005 and WNV NY99-4132 strains were used for serologic assays. CbaAr-4005strain of SLEV was isolated from *Culex quinquefasciatus* mosquitoes collected in Córdoba during the human encephalitis outbreak of 2005 [5]. NY99-4132 strain of WNV was obtained from the brain of an American Crow (*Corvus brachyrhynchos*) collected in New York during 1999. Viral stocks were obtained from infected Vero cell monolayers harvested on day 7 and 5 post-inoculation for SLEV and WNV, respectively.

### Serological assays and data interpretation

Avian serum samples were screened for the presence of WNV- and SLEV-reactive antibodies by the plaque-reduction neutralization test (PRNT). A suspension of approximately 100 plaque-forming units (PFU) of virus in 75 μL of MEM was added to an equal volume of diluted avian serum, bringing the final serum dilution to 1:20. The mixture was incubated for 1 hour at 37°C. Vero cell monolayers grown in 24-well culture plates (Costar, Cambridge, MA, USA) were inoculated with 0.1 mL of the serum-virus mixture and incubated for 1 hour at 37°C, 5% CO_2_. Cells were overlaid with an initial 1 mL of 0.5% agarose in M-199 medium supplemented with 350 mg/L sodium bicarbonate, 29.2 mg/L L-glutamine, and antibiotics (penicillin, streptomycin, gentamycin, and amphotericin B). After 3 and 6 days of incubation with WNV and SLEV, respectively, cultures were overlaid again with the same nutrient–agarose mix but also containing 0.004% neutral red for staining and visualization of viral plaques. Plaques were counted the following day. Serum samples that neutralized > 80% of the challenge virus (relative to a serum-free control) were selected for further titration against both WNV and SLEV. Flavivirus titers of serum samples that tested positive in screen tests were determined as follows. Seven serial two-fold dilutions of serum in MEM were prepared beginning with a dilution of 1:10. Virus mixtures were added as described above, resulting in final serum dilutions of 1:20, 1:40, 1:80, 1:160, 1:320, 1:640 and 1:1280. Endpoint titers were assigned as the reciprocal of the greatest dilution in which >80% neutralization of the challenge virus was achieved. Experiments that evaluated cross reaction among SLEV and WNV in heterologous inoculation scenarios in Common Quail (*Coturnix coturnix*) indicated no cross reaction among SLEV and WNV (Contigiani MS personal communication).Based on this evidence, plus studies by Patiris et al. [11] and Ledermann et al. [12], we considered all serum samples with antibody titers higher than 20 positive. Therefore samples with titers higher than 20 for both viruses were considered as multiple heterologous infections.

### Statistical analysis

Infection prevalence with 95% confidence intervals were calculated for both viruses. Infection proportions within the year and month of sampling were compared using the Fisher exact test or Pearson chi-square test. P-values were considered significant at a threshold of α = 0.05. The homology between spatial activity for SLEV and the bird community was explored by means of a Procrustes analysis [13]. First, we selected a subset of 6 species that accounted for the majority of captures (51%) and 70% of SLEV seroprevalence, which included Eared Dove (*Zenaida auriculata*), Picui Ground-Dove (*Columbina picui*), Rufous Hornero (*Furnarius rufus*), House Sparrow (*Passer domesticus*), Great Kiskadee (*Pitangus sulphuratus*) and Bay-winged Cowbird (*Agelaioides badius*). These 6 species were represented in all sampled sites. Abundance and absolute prevalence matrix were built and data exploration showed the need to remove VP from the dataset (due to double zeros abundance). These frequency matrices were used to produce unconstrained ordinations analysis with chi-square distance (community assemblage matrix) and Non-metric multidimensional scaling (NMDS) (SLEV seroprevalence matrix). Finally, the ordination configuration homology was estimated by a Procrustes analysis and its statistical significance was calculated after 9999 permutations [13].

All analyses were run using vegan and core packages within the R platform [14,15,16].

In order to explore the effect of new species detected as infected on the overall avian seroprevalence among the study periods, we compared the proportion of infected birds of the selected species (same subset as used for the spatial analysis) vs. the proportion of infected birds of the non-selected species, for a given period.

## Results

A total of 980 birds were sampled for detection of SLEV- and WNV-neutralizing antibodies. Analyzed sera belonged to 65 species and 27 families of free-ranging birds. SLEV seroprevalence in birds increased 11-fold from 2004 to 2005 (Table 1). Although the difference in seroprevalence observed between years was significant (Fisher exact test, p <0.001), no significant difference was detected between months within each year (2004, χ2 = 1.51, df = 3, p = 0.68; 2005, χ2 = 0.44, df = 2, p = 0.80). During January-April 2004 only 3 birds tested positive for SLEV-neutralizing antibodies (0.7%). SLEV activity was found in 3 resident (i.e. non-migratory) bird species: Rufous Hornero (2.0%, 1/49), Bay-winged Cowbird (8.3%, 1/12) and Great Kiskadee (1.2%, 1/80). These three seropositive individuals were distributed among three of the four study sites (Fig 1). One of these (Rufous Hornero) seroconverted from January to March (Table 2). Of 14 other recaptured birds in 2004, none seroconverted. In the second period (January-March 2005), an overall SLEV seroprevalence of 7.7% (42/543) was registered, with seropositive birds detected at all four study sites. Multiple infections for SLEV and WNV were detected in 6 serum samples (1.1%).Neutralizing antibody titer ranged from 40 to 1280, with titers of 40 and 160 the most frequently detected. SLEV seroprevalence increased between years in all three paired-year sites, showing significance differences between 2004 and 2005 (BG: χ2 = 136; p<0.001; CS: χ2 = 58; p = 0.01 and VP: χ2 = 56.35; p = 0.01) (Fig 1). Differences were also observed among sites within 2005 (χ2 = 24.53; df = 3; p = 0.006).

**Table 1:** St. Louis encephalitis virus-neutralizing antibody prevalence among all bird species combined, by months and years, Córdoba, Argentina.

*Month*	*2004*		*2005*	
	*Pos/Total*	*%[CI]*	*Pos/Total*	*%[CI]*
January	1/110	0.90[0.05–5.69]	12/145	8.28[4.54–14.32]
February	0/137	0.00 [0.00–3.40]	15/178	8.43[4.96–13.77]
March	1/76	1.31[0.07–8.11]	15/220	6.81[4.00–11.21]
April	1/114	0.88[0.05–5.50]	ND	ND
Total	3/437	0.69[0.18–2.17]*	42/543	7.73[5.69–10.39]*

*p-value<0.001.

ND: no data available.

CI: 95% confidence interval.

**Table 2:** Seroprevalence among free-ranging birds collected in Córdoba city during 2005 and tested by PRNT for St. Louis encephalitis- and West Nile virus-reactive antibodies.

*Family*	*Species*	*Migratory Status*	*SLEV*		*WNV*	
			*Pos/Tested*	*%[CI]*	*Pos/Tested*	*%[CI]*
Falconidae						
	American Kestrel (*Falco sparverius*)	Resident	0/2	0 [0–65.76]	1/2	50 [9.45–90.55]
	ChimangoCaracara(*Milvago chimango*)	Resident	1/1	100 [20.65–100]	0/1	0 [0–79.35]
Columbidae						
	Eared Dove (*Zenaidaauriculata*)	Resident	4/33	12.12 [4.82–27.33]	0/33	0 [0–10.43]
	Picui Ground-Dove (*Columbinapicui*)	Resident	10/65	15.38 [8.58–26.06]	0/65	0 [0–5.58]
Picidae						
	Green-barred Woodpecker (*Colaptesmelanochloros*)	Resident	2/6	33.33 [9.68–70]	0/6	0 [0–39.03]
Furnariidae						
	Brown Cacholote (*Pseudoseisuralophotes*)	Resident	2/7	28.57 [8.22–64.11]	0/7	0 [0–35.43]
	Rufous Hornero (*Furnariusrufus*)	Resident	2/54	3.7 [1.02–12.54]	5/54	9.26 [4.02–19.91]
Tyrannidae						0 [0–19.36]
	Elaenia spp. (*E*. *albiceps*/*parvirostris*)	Migratory	1/16	6.25 [1.11–28.33]	0/16	
	Great Kiskadee(*Pitangussulphuratus*)	Resident	12/91	13.19 [7.71–21.65]	1/91	1.10 [0.19–5.96]
	Vermilion Flycatcher (*Pyrocephalusrubinus*)	Migratory	1/8	12.50 [2.24–47.09]	0/8	0 [0–32.44]
Turdidae						
	Creamy-bellied Thrush (*Turdusamaurochalinus*)	Resident	1/20	5 [0.89–23.61]	0/20	0 [0–16.11]
Thraupidae						
	Black-and-rufous Warbling-Finch (*Poospizanigrorufa*)	Resident	1/7	14.29 [2.57–51.31]	0/7	0 [0–35.43]
	Black-crested Finch (*Lophospinguspusillus*)	Resident	1/1	100 [20.65–100]	0/1	0 [0–79.35]
Emberizidae						
	Rufous-collared Sparrow (*Zonotrichiacapensis*)	Resident	1/9	11.11 [1.99–43.5]	0/9	0 [0–29.91]
Icteridae						
	Bay-winged Cowbird (*Agelaioidesbadius*)	Resident	1/34	2.94 [0.52–14.92]	0/34	0 [0–10.15]
Passeridae						
	HouseSparrow(*Passerdomesticus*)	Resident	2/51	3.92 [1.08–13.22]	1/51	1.96 [0.35–10.30]
**TOTAL**			42/543	7.73 [5.77–10.29]	8/543	1.47 [0.75–2.88]

**Seronegative species belonging to seropositive families:** Columbidae: *Patagioenasmaculosa*(1), *Leptotilaverreauxi*(2); Furnariidae: *Asthenesbaeri*(1), Tyrannidae: *Knipolegusaterrimus*(1), *K*. *striaticeps* (1), *Machetornisrixosus (6)*, *Myiarchustyrannulus*(1), *Pseudocolopteryxacutipennis*(2), *Serpophagasubcristata*(2), *Tyrannus savanna* (1), *Pachyramphuspolichopterus*(1); Non identified (9); Turdidae: *Turdusrufiventris*(1); Thraupidae: *Saltatoraurantirostris* (2), *Pipraeideabonariensis*(1), *Coryphospinguscucullatus*(1), *Embernagraplatensis* (1), *Poospiza ornate* (1), *Sicalisflaveola*(12), *S*. *luteola*(1), *Sporophilacaerulescens* (10); Emberizidae: *Rhynchospizastrigiceps*(1), *Ammodramushumeralis*(1); Icteridae: *Chrysomusruficapillus* (2), *Molothrusrufoaxillaris*(9), *M*. *bonariensis*(10), *Agelasticusthilius* (3), *Chrysomusruficapillus*(2), Non identified (1).

**Seronegative families and species**: Charadriidae: *Vanelluschilensis*(1); Psittacidae: *Myiopsittamonachus* (2); Cuculidae: *Coccyzusmelacoryphus* (4); Bucconidae: *Nystalusmaculatus*(1); Thamnophilidae: *Taraba major (3);* Cotingidae: *Phytotomarutila* (9); Vireonidae: *Cyclarhisgujanensis*(1); Troglodytidae: *Troglodytes aedon (6);* Mimidae: *Mimustriurus (21)*; Parulidae: *Geothlypisaequinoctialis (2);* Fringillidae: *Spinusmagellanica (4)*

In 2005, the number of SLEV-seropositive bird species increased from 3 in the previous year to 15 (Table 3). The avian families with the highest seroprevalence for SLEV-neutralizing antibodies were: Columbidae (14.0%; 14/101); Tyrannidae (10.1%; 14/138); Furnariidae (6.3%; 4/63); Thraupidae (5.5%; 2/36); Turdidae (4.5%; 1/22); Passeridae (3.9%; 2/51); and Icteridae (1.7%; 1/59). The seroprevalence by bird species ranged between 0% and 28.6%. Highest values were observed in Brown Cacholote (*Pseudoseisura lophotes*, 28.6%; 2/7) and Picui Ground-Dove (15.4%; 10/65). Two out of 25 recaptured birds seroconverted for SLEV during the January-March 2005 period (Table 2).

**Table 3:** St. Louis encephalitis and West Nile virus seroconversion events in recaptured resident birds during 2004 and 2005.

*Species*	*Site*	Months sampled(*)	*PRNT80titer*				*Virus*
			SLEV		WNV		
			1st sample	2nd sample	1st sample	2nd sample	
Rufous Hornero	CS	Jan-Mar '04 (56)	<20	40	<20	<20	**SLEV**
Totalrecapturedindividuals 2004							**15**
Bay-wingedCowbird	BG	Jan-Feb '05 (31)	<20	80	<20	<20	**SLEV**
Great Kiskadee	BG	Jan-Mar '05 (57)	<20	640	<20	<20	**SLEV**
Great Kiskadee	BT	Feb-March'05 (54)	<20	<20	<20	40	**WNV**
Rufous Hornero	CS	Jan-Feb '05 (34)	<20	<20	<20	640	**WNV**
Rufous Hornero	CS	Jan-Mar '05 (71)	<20	<20	<20	640	**WNV**
Rufous Hornero	BG	Jan-Mar '05 (58)	<20	<20	<20	80	**WNV**
Total recaptured individuals 2005							**25**

*Days between date capture-recaptured

**Fig 2:**
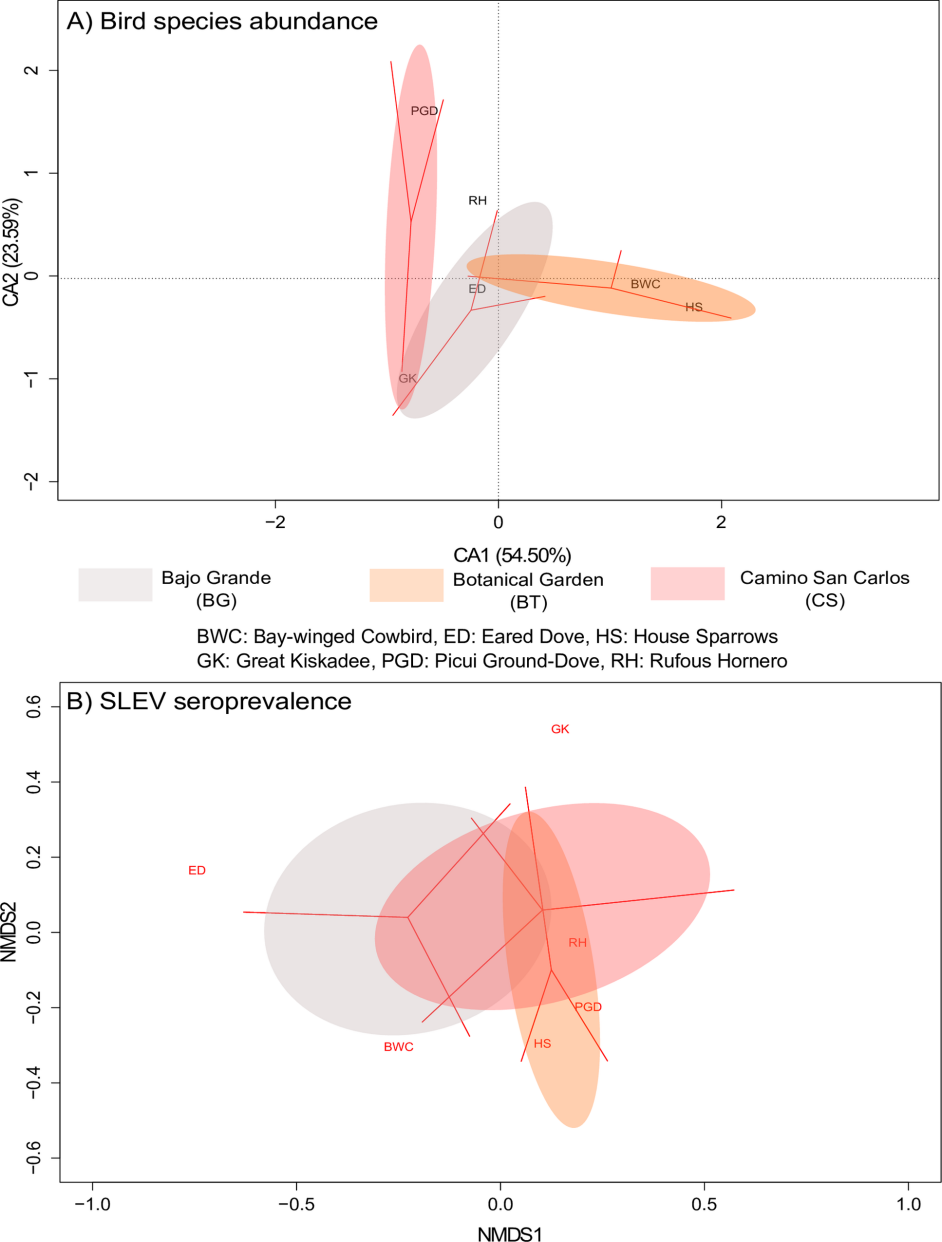
Ordination analyses based on a subset of 6 bird species selected collected and tested in three sites of Córdoba city from January to March 2005. A) Correspondence analysis ordination bi-plot for bird species abundance derived from frequency of capture in mist-nets. For each axis, the amount of variation explained as a part of the total variation in the model is shown. B) Non-metric multidimensional scaling (NMDS) analysis ordination bi-plot for SLEV seroprevalence. Colored areas represent amount of variation for analyzed variables.

Bird community assemblage ordination showed pairwise overlapping (BG-CS, BG-BT) total inertia 0.62. Two first axes explained 78% of the variation (Fig 2A). Great Kiskadee is a common element in the BG-CS paired cluster. Picui Ground-Dove, Eared Dove and Bay-winged Cowbird/House Sparrow abundances are key species in the ordination of CS, BG and BT respectively. Rufous Hornero was common among all sites analyzed. Two convergent solutions were found after six iterations running NMDS analyses (stress: 0.1267; non-metric fit R^2^ = 0.9840; RMSE = 2.77^−5^). SLEV prevalence ordination shows a complete overlapping among analyzed sites suggesting a similar composition of infected bird species (Fig 2B). In site BG, infected individuals of all six selected species were detected. Infected Shiny Cowbird (*Molothrus bonariensis*) was only detected here. Sites BT and CS share the absence of infection in Shiny Cowbird, Eared Dove and Rufous Hornero (Fig 2A). Procrustes test was not significant (m^2^ = 0.7775; t_0_ = 0.4717; p-value = 0.3456) and failed to detect an association pattern between SLEV prevalence and bird abundance by sites.

**Fig 3:**
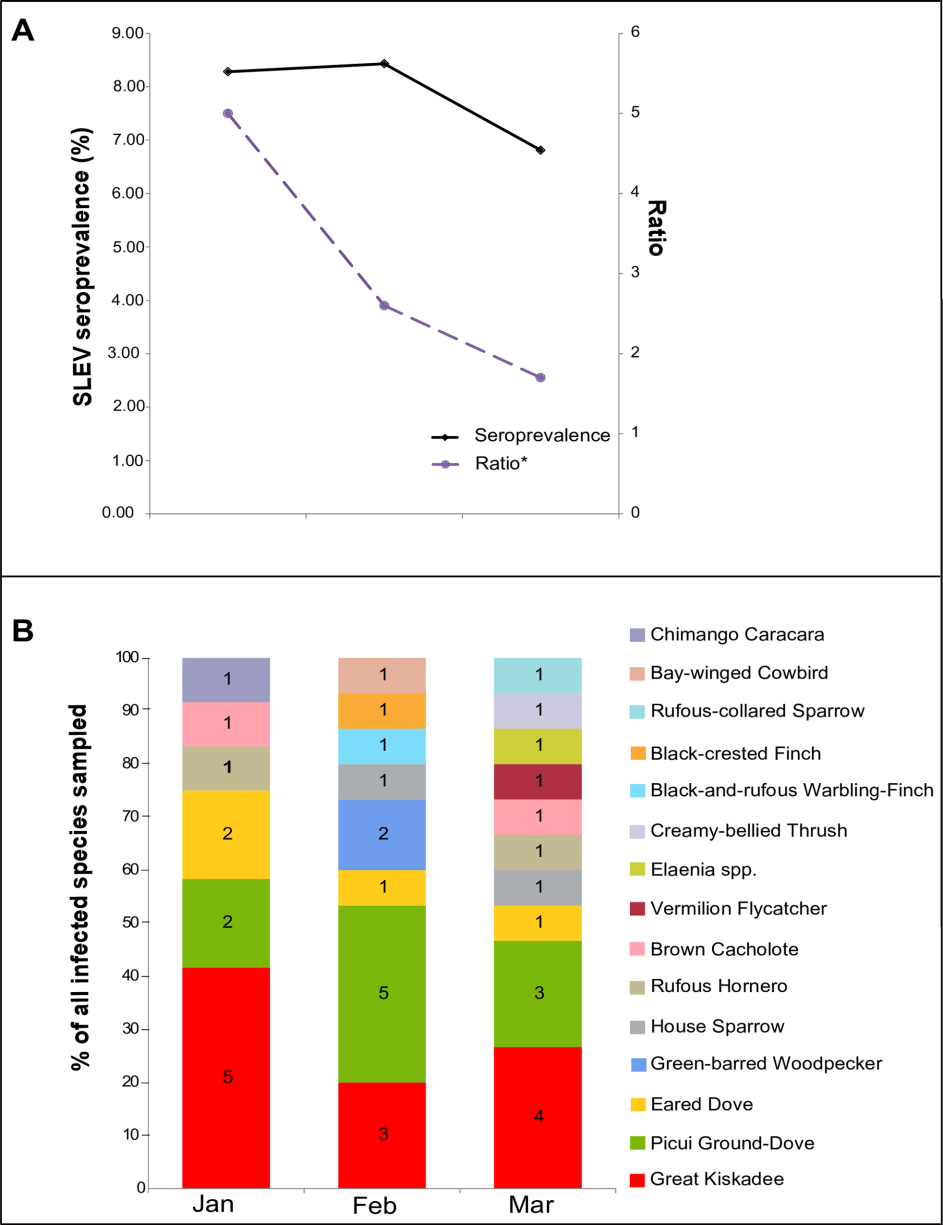
St. Louis encephalitis virus host infection dynamics during January-March 2005. A) SLEV infection prevalence and the ratio of selected vs non-selected infected bird species. B) Species composition of seropositive birds for SLEV.

During 2005, whereas the SLEV seroprevalence among birds appeared to be relatively stable, with no significant change among sampling months, the number of infected bird species increased throughout the sampling period. Moreover, the relative importance of the 6 selected species used in the spatial analysis diminished from January to March as more species were detected as seropositive each month (Fig 3A). The monthly infected bird species richness increased by 67% from January to March (Fig 3B). Only Picui Ground-Dove, Great Kiskadee and Eared Dove were detected as seropositive in all three months surveyed in 2005. Picui Ground-Dove and Great Kiskadee together contributed the largest number of infected individuals by month (approximately 50% of all seropositive birds each month).

No WNV-neutralizing antibody was detected during January–April, 2004, among 437 bird sera analyzed. However, during January–March, 2005, the WNV seroprevalence observed was 1.5% (8/543, binomial 95% CI: 0.6–2.9%). Highest WNVseroprevalence was observed in March (2.3%; 95%CI = 1.7% - 9.0%;5/125) compared to January (0.9%;95% CI = 0.2% - 4.8%; 1/114) and February (0.5%; 1/209; 95%CI = 0.1% - 2.7%).WNV seropositive birds were observed in BG (1.3%; 2/160; 95%CI = 0.3% - 4.4%), BT (1.1%; 2/176; 95%CI = 0.3% - 4.1%) and CS (2.0%; 3/151; 95%CI = 0.7% - 5.7%). Only four out of 52 bird species were positive for WNV-neutralizing antibodies: American Kestrel (*Falco sparverius*); Rufous Hornero; House Sparrow; and Great Kiskadee (Table 2). Four WNV seroconversion events were registered out of 25 opportunities in 2005, most of them in Rufous Hornero (Table 3).

## Discussion

Arbovirus activity is influenced in a complex manner by several biological and environmental factors [17]. As in North America, it appears thatcertain birds play an important role in the amplification of SLEV during outbreaks of human disease.

Our study indicated that a high proportion (99.3%) of local birds were susceptible to SLEV infection immediately prior to the 2005 outbreak, indicating that the vertebrate host population was primed to amplify SLEV. Another biologic factor that may have promoted the SLEV outbreak could be the sustained increase in abundance of the main vector *Culex quinquefasciatus* which has been documented elsewhere [18,19]. In temperate areas, arbovirus transmission is highly influenced by mosquito vector abundance [20,21]. In urban sites of central Argentina, SLEV is mainly transmitted by *Culex quinquefasciatus* [22] and potentially also by *Culex interfor* [5]. These mosquitoes’ populations have two annual peaks: a small one in late spring-early summer (November-December) and a large one in late summer-early fall (February-March) [18,19]. Based on this vector pattern one would expect the presence of two peaks of SLEV activity, which indeed was observed during this human encephalitis outbreak [1]. The high avian SLEV seroprevalence detected in January, followed by no discernable increase in antibody prevalence during February and March suggests that much of the transmission to birds had already occurred by January 2005 or earlier, which corresponds with the earlier peak of vector abundance. Some transmission continued in birds after January as evidenced by two seroconversion events (Table 3) and an expanding number of seropositive bird species (Fig 3B).

The apparent reduction in avian seroprevalence for SLEV in March 2005 can be explained in several ways: 1. By March, conditions for SLEV amplification among birds were no longer favorable, and the detectable seroprevalence dropped as a consequence of normal bird movements (emigration of seropositive individuals and immigration of seronegative individuals from regions where SLEV was not active); 2. Diminishing detectability of SLEV antibodies as birds aged and their antibodies, derived from infections several months earlier, began to wane to undetectable levels. SLEV antibodies in some birds infected during December to January could become undetectable in March due to diminishing neutralizing antibody titers below the detection threshold (20) [23,24].

SLEV was found distributed in a variety of environments (urbanized neighborhoods as well as patches of native habitat) throughout the city of Córdoba, indicating that ecological requirements for its maintenance are present. However, variation in the force of viral transmission among sites was observed. The highest activity was registered in the eastern peri-urban site (BG). This area contains large polluted water reservoirs and trash dumps, which support an abundant population of *Culex quinquefasciatus*. These landscape elements agree with those associated with risk of human infection identified by Vergara Cid et al. [25]. Differences in transmission among sites would be due to fine scale differences in populations of vectors and vertebrate hosts.

Infected bird species found during the SLEV epidemic period belong to the same families (Columbidae, Furnariidae, Icteridae, Tyrannidae) as those detected during enzootic periods further north in Argentina [6]. Presumably, a strain of SLEV would utilize similar vector and vertebrate host species for its maintenance/amplification throughout its geographic range. Hence, spatiotemporal variation in transmission would result from differing ecological features such as vector and vertebrate host abundance and distribution, habitat use and population dynamics. Within the epidemic period, we observed an increase in the number of SLEV-infected bird species between months (Fig 3). Whether this was due to a change in transmission ecology (i.e. a shift of transmission to novel amplifier hosts) or spillover to tangential avian hosts remains unknown. However, infected individuals of Picui Ground-Dove and Great Kiskadee were reliably present at all sampling sites.

Multivariate analysis used in the geospatial analyses compared the bird species composition and SLEV seroprevalence among the study sites, but failed to detect a clear separation pattern. In our study, avian community assemblage varied among sites (Fig 2A) yet SLEV seroprevalence ordination overlapped with all sites. The overlapping pattern observed in SLEV seroprevalence could be explained by the presence of identical/similar mosquito vector(s) with similar host preferences. Such was the case for WNV in the eastern USA where viral activity is driven by mosquito host preference [26]. Although WNV and SLEV share ecological requirements, this hypothesis must be corroborated for SLEV.

Eared Dove and Picui Ground-Dove (Columbidae), Great Kiskadee (Tyrannidae) and Brown Cacholote (Furnariidae) had high seroprevalence (>12%), are widely dispersed and are abundant in several ecosystems (urban and rural habitats). Moreover, seropositive individuals of Picui Ground-Dove and Great Kiskadee were frequently detected during the epidemic period (Fig 3). These features make these species candidates for amplifying hosts of SLEV. Experimental infections of adult Eared Dove demonstrated this species’ competence to infect a local strain of *Culex quinquefasciatus* with the enzootic 78V-6507 strain of SLEV [27]. Another interesting species is Rufous Hornero. Although its infection prevalence was low (3.7%), it is abundant in urban and rural areas. Since it is highly territorial and easy to collect with mist nets, and it is frequently infected with both SLEV and WNV, it could represent an important amplifier as well as a good sentinel for flavivirus transmission activity. The House Sparrow is the main SLEV host in largely urban areas of the USA[3]. However, previous reports from Argentina highlighted the absence of SLEV-infected House Sparrow (more than 200 sera analyzed) and questioned its role as amplifying host for SLEV in Argentina [6]. For the first time in South America, our study detected SLEV-infected individuals of House Sparrow (seroprevalence<5%). Host competence assays carried out with North American House Sparrow and SLEV strains isolated in South America suggested a poor amplification potential for House Sparrow [28,29]. However, without data on host competence derived from infection studies matching Argentinean strains of SLEV with a sympatric population of House Sparrow, we cannot speculate regarding its role as an amplifier of SLEV in Argentina.

The first evidence of autochthonous circulation for West Nile virus (WNV) in the American continent was detected in 1999 in New York City [30]. Thereafter, it rapidly spread throughout the New World [31,32]. In Argentina this virus caused epizootic events in equines [7]. Molecular characterization indicated that the introduced strain in Argentina belongs to the I2 lineage (NY99 strain cluster) [33]. A serosurvey of free-ranging birds indicated widespread transmission, with no evidence for disease detected in Argentinean birds [8]. The earliest detection of WNV-neutralizing antibodies in birds from Argentina was January 2005, indicating that WNV circulation in this country had begun by the end of 2004. In the present study, we document four birds that seroconverted to WNV sometime between January and March 2005. One of them was negative in January and had seroconverted to positive 34 days later in February. Host competence assays carried out in urban birds have indicated that the Picui Ground-Dove is competent to amplify WNV [34]. However none of 65 representatives of this species was found to have been infected by WNV in Córdoba.

In summary, we showed that in 2005, both SLEV and to a lesser extent WNV circulated in a variety of avian species. Seroprevalence combined with avian abundance may be used to identify candidate amplifier hosts. Competence studies are needed to verify the importance of these candidates as amplifiers, as well as vector feeding studies to confirm vector-amplifier host contact. Eared Dove, Picui Ground-Dove and Great Kiskadee are strong candidates to amplify SLEV. Rufous Hornero and Brown Cacholote are also frequently infected and may be useful sentinels because of their local residence, territoriality and ease of capture. Rufous Hornero may be an important maintenance host for WNV in central Argentina.
